# Eurosurveillance 5^th^ scientific seminar on 30 November at ESCAIDE - 20 years of communicating facts and figures in a changing world

**DOI:** 10.2807/1560-7917.ES.2016.21.46.30401

**Published:** 2016-11-17

**Authors:** Eurosurveillance editorial team

**Affiliations:** 1 European Centre for Disease Prevention and Control (ECDC), Stockholm, Sweden

On the occasion of our 20-year anniversary, the 5^th^*Eurosurveillance* Scientific Seminar will have ‘20 years of communicating facts and figures in a changing world’ as its topic.

Featuring speakers Professor David Heymann, Head of the Centre on Global Health Security at Chatham House, London, and Chairman of Public Health England, UK, and Professor Lawrence Madoff, Director, Division Of Epidemiology And Immunization, Massachusetts Department of Public Health and Editor of ProMED-mail (the programme for Monitoring Emerging Diseases), and moderated by Professor Panayotis Tassios, Associate editor of *Eurosurveillance,* the seminar is held at the same venue as ESCAIDE to ensure that as many participants as possible can use the opportunity to listen to the speakers.

For the full programme, please go to the *Eurosurveillance* homepage.

